# Overall Survival after Radiotherapy for Brain Metastases According to ECOG Status—A Prospective Study of 294 NSCLC Patients

**DOI:** 10.3390/cancers16081486

**Published:** 2024-04-12

**Authors:** Astrid Telhaug Karlsson, Marianne Jensen Hjermstad, Nina Aass, Eva Skovlund, Stein Kaasa, Olav Erich Yri

**Affiliations:** 1 Regional Advisory Unit for Palliative Care, Department of Oncology, Oslo University Hospital (OUH), 0450 Oslo, Norway; mariajhj@medisin.uio.no (M.J.H.); naa@ous-hf.no (N.A.); stekaa@ous-hf.no (S.K.); olavy@ous-hf.no (O.E.Y.); 2 European Palliative Research Centre (PRC), Oslo University Hospital (OUH), 0450 Oslo, Norway; 3 Institute of Clinical Medicine, University of Oslo, 0424 Oslo, Norway; 4 Department of Public Health and Nursing, Faculty of Medicine and Health Services, NTNU—Norwegian University of Science and Technology, 7491 Trondheim, Norway; eva.skovlund@ntnu.no

**Keywords:** non-smallcell lung cancer, brain metastases, radiotherapy, performance status, overall survival

## Abstract

**Simple Summary:**

Non-smallcell lung cancer patients frequently develop brain metastases. Most patients are treated with radiotherapy, but the potential benefit from radiotherapy in patients with poor performance status is uncertain. Consequently, the risk of futile treatment is high. This study provides knowledge to improve the treatment decision-making process. We report a prospective study of 294 patients treated with radiotherapy for brain metastases in 2017–2021, focusing on the correlation between performance status and survival. Our data support that patients with ECOG performance status 3–4 should not be offered radiotherapy, as they are likely to have very short expected survival. Also, if not candidates for surgery or stereotactic radiotherapy, patients with ECOG 2 have an expected survival equal to those with ECOG 3–4, especially if extra-cranial metastases are uncontrolled. Therefore, these patients should be offered the best supportive care rather than WBRT to improve symptoms and preserve quality of life near the end of life.

**Abstract:**

Up to 40% of non-smallcell lung cancer (NSCLC) patients develop brain metastases (BMs). The potential benefits of radiotherapy (RT) in patients with poor performance status (PS) are questionable, with considerable risk for futile treatment. We analyzed overall survival after initial radiotherapy in NSCLC patients with BMs, focusing on the relationship between PS and survival after RT. This study reports a prospective observational study including consecutive 294 NSCLC patients with first-time BMs. Overall survival (OS) was calculated from the start of RT to death or last follow-up (1 June 2023). Overall, in the 294 included patients (median age 69 years), the median OS was 4.6 months; 2.5 months after WBRT (*n* = 141), and 7.5 months after SRT (*n* = 153). After WBRT, mOS was equally poor for patients with ECOG 2 (1.9 months) and ECOG 3–4 (1.2 months). After SRT, mOS for patients with ECOG 2 was 4.1 months; for ECOG 3 patients, mOS was 4 1.6 months. For NSCLC patients with ECOG 2 diagnosed with BMs who are not candidates for surgery or SRT, WBRT should be questioned due to short survival.

## 1. Introduction

Lung cancer is the second most common cancer both in Norway and worldwide [1,2]. At least 40% of patients with non-smallcell lung cancer (NSCLC) are diagnosed with brain metastases (BMs) during the disease trajectory [3,4,5]. The incidence of BMs is increasing following more frequent screening [6], more accurate brain imaging with magnetic resonance imaging (MRI), and prolonged survival with new systemic treatment options [7].

Over time, standard-of-care treatment for NSCLC BMs has changed, with more use of stereotactic radiotherapy (SRT) and new systemic treatments. Targeted therapies have intracerebral response rates of 40–80%, resulting in prolonged survival in patients with driver mutations [8], and there are limited but promising data on the effect of immunotherapy in NSCLC patients with BMs [9]. For patients with symptoms due to high intracerebral pressure or the need for a biopsy, surgery is still the recommended treatment, regardless of systemic therapy options [10].

However, radiotherapy (RT) remains the cornerstone of BM treatment in the majority of NSCLC patients. Historically, whole brain radiotherapy (WBRT) has been the treatment of choice, but during the last two decades, SRT has become the favored treatment approach in patients with 1–4 BMs. This is due to RCTs reporting no difference in overall survival, but less cognitive decline with SRT alone [11,12]. Additionally, the QUARTZ study found no difference in median overall survival (mOS) comparing best supportive care (BSC) to WBRT in NSCLC patients who were not candidates for SRT or surgery [13]. Consequently, current treatment guidelines do not recommend RT to patients with Karnofsky Performance Status (KPS) ≤ 50, nor to patients with KPS < 70 without systemic therapy options [10]. These patients should rather be given BSC alone. However, WBRT is still widely administered to lung cancer patients with poor performance status, i.e., ECOG 3–4 [14,15].

Futile treatment is defined as treatment without any benefit to the patient [16]. To better identify patients who will benefit from treatment in terms of survival and/or symptom relief, prognostic tools like Recursive Partitioning Analysis (RPA) and Lung Graded Prognostic Assessment (GPA) were developed [17,18]. An updated version of RPA (U-RPA) was published in 2023 [19]. Such tools are frequently used for stratification in clinical trials but are not systematically used in real life [14].

The potential benefits of radiotherapy (RT) in patients with poor performance status (PS) are questionable, with considerable risk for futile treatment [10]. In clinical practice, an ECOG status score of 2 is commonly regarded as a reasonably good performance status that justifies the start of RT. However, few prospective, real-life studies have investigated the association between overall survival, ECOG score, and RT as first-line treatment for BMs. 

Brain Metastases in Norway—A Prospective Cohort Study (NCT03346655), was a prospective population-based study that collected data from an un-selected, real-life patient cohort with first-time BMs, including treatment patterns and outcomes after treatment. The overall goal was to provide knowledge to improve treatment decision making and to avoid futile treatment at the end of life. This paper reports on the NSCLC cohort of that study, presenting overall survival after first-time RT in relation to ECOG performance status. The relevance of Lung GPA and U-RPA in an unselected real-life cohort is also analyzed. 

## 2. Materials and Methods

### 2.1. Study Design

A prospective observational study was conducted including consecutive patients diagnosed with first-time BMs from solid cancers from November 2017 to March 2021. Given the observational, real-life design, a pragmatic, practice-based approach was taken regarding BM diagnostics, treatment decisions, choice of treatment modality, radiation dosage, and RT planning. A central review was not performed for study purposes. 

### 2.2. Identification

Patients were diagnosed with BMs after CT and/or MRI due to screening, routine follow-up procedures, or clinical symptoms. Diagnostics were carried out per protocol and practice at the different hospitals in the healthcare region that referred patients for RT. Patients were included at oncological centers in five hospitals in Norway but were referred to radiotherapy from all local hospitals in the two healthcare regions. They were identified by study personnel according to schedules/timetables or referrals and included immediately before the start of radiotherapy. For the NSCLC cohort, the following inclusion criteria applied: histologic, clinical, or radiological evidence of NSCLC, BMs verified by CT and/or MRI, age >18 and ability to comply with study procedures, and ability to understand and provide written informed consent in Norwegian. Exclusion criteria were primary brain tumors, primary hematological malignancies, previous diagnosis and/or treatment of BMs, and inability to produce written consent. 

### 2.3. Patient Characteristics

Patient and disease characteristics were collected from electronic medical records in accordance with a study-specific checklist of relevant variables defined by experienced oncologists and researchers in the study. The status of extracranial metastases (ECMs) was defined according to the most recent CT scan at the time of BM diagnosis as “controlled” (no/stable ECM) and “uncontrolled” (progressive/unknown or synchronous ECM and BM). Complete follow-up data were collected from medical records at the respective local oncological centers. The number of BMs was based on diagnostic brain CT if no brain MRI was available. In our analysis, patients were categorized into the “WBRT group” or “SRT group” according to their first RT modality. For survival analysis, OS was calculated from the start of radiotherapy to the date of death or last follow-up (1 June 2023). Overall survival <3 months was defined as poor survival.

### 2.4. Treatment

The choice of radiotherapy modality, SRT or WBRT, was at the discretion of the treating physician. In general, SRT is indicated for 1–4 BMs < 4 cm in diameter, with gross tumor volume defined by a visible tumor on planning MRI with a margin of 1–2 mm. WBRT is mainly used in patients with ≥5 BMs, but performance status, extracranial disease status, and patient preferences are other factors considered in the treatment selection. The radiotherapy was performed on a linear accelerator system (LINAC). Whole brain radiotherapy was performed by two lateral opposed fields, and SRT was performed using the VMAT/IMRT technique. None of the patients received gamma knife treatment.

### 2.5. Lung GPA and Updated RPA

The predefined checklist included all the variables necessary for Lung GPA and U-RPA. The rating of the ECOG performance scale was at the discretion of the treating physician. If performance status was missing in the medical records, an experienced nurse or lung oncologist estimated the ECOG score at study inclusion. As ECOG performance status scale is commonly used in Norway, we converted ECOG to KPS as follows: ECOG 0 = KPS 100–90, ECOG 1 = 80–70, ECOG 2 = KPS 60–50, ECOG 3 = KPS 40–30, ECOG 4 = KPS, and ECOG 5 = KPS 0 [20]. For both prognostic tools, patients were categorized into prognostic groups or classes according to summarized scores or prognostic factors [16,17]. The updated RPA (U-RPA) sub-classifies the patients in class 2 according to their KPS, 2A (KPS 90–100/ECOG 0) and 2B (KPS 70–80/ECOG 2–1) [19]. To compare estimated mOS based on Lung GPA and U-RPA, actual mOS in our patient cohort was calculated from the date of BM diagnosis. According to the authors, patients with Lung GPA scores 0.0–1.0 (predicted mOS 6 months for adenocarcinoma and mOS 2 months for non-adenocarcinoma) or U-RPA class 2B and 3 (predicted mOS 7.6 months and 3.3 months, respectively) are considered to have a poor prognosis.

### 2.6. Statistical Analysis

Standard descriptive statistics were used for patient characteristics. Overall survival was analyzed by the Kaplan–Meier estimator supplemented by the log-rank test. Patients still alive were censored at the time of the last follow-up (1 June 2023). Uni- and multivariable analyses were performed by the Cox proportional hazards model to determine which clinical factors were associated with longer survival. All analyses were performed by SPSS Statistics 28 (IBM Corp., Armonk, NY, USA). Non-overlapping 95% CI represents a *p*-value of <0.05 [21].

### 2.7. Ethical Considerations

This study received ethical approval from the Regional Committees of Medical and Health Research Ethics in each health region (reference no. 2017/1358). Authorization for data access was approved by the Data Protection Agency at each hospital. All data are stored and handled in accordance with General Data Protection Regulations (GDPR). Patients received oral and written information about the study and signed a written consent.

The STROBE guidelines for cohort studies were followed (Table S1) [22].

## 3. Results

### 3.1. Characteristics

In total, 294 patients were included (median age 69 years (range 35–92); 51% female). At the last follow-up (1 June 2023), 42 patients (14%) were still alive. The median follow-up time was 4.5 months (range 0.2–60.6). Fifty-two percent (*n* = 153) of the patients had a synchronous diagnosis of BMs and lung tumor. Overall, 30% (*n* = 89) had one BM and 35% (*n* = 102) had ≥5 BMs. Extracranial metastases (ECMs) were uncontrolled in 58% (*n* = 170) of the patients; 29% (*n* = 85) were categorized as ECOG 2 and 16% (*n* = 47) as ECOG 3–4, respectively (Table 1).

### 3.2. Treatment

**Figure 1:**
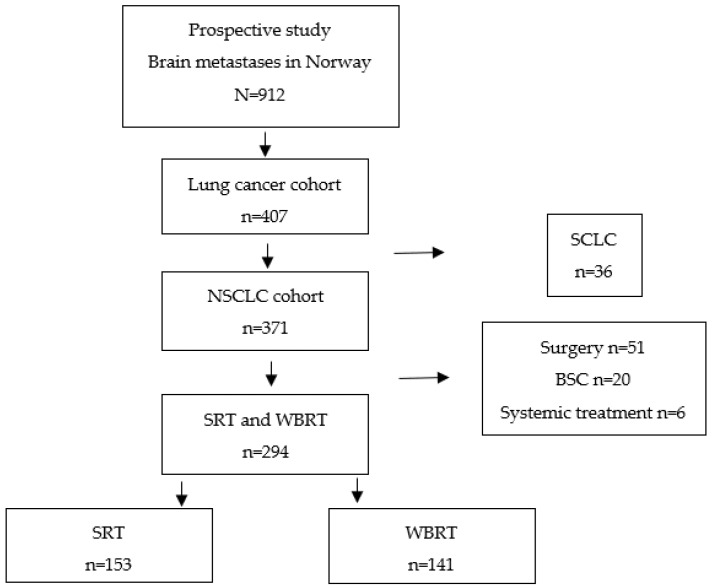
Treatment breakdown. Non-smallcell lung cancer (NSCLC), stereotactic radiotherapy (SRT), whole brain radiotherapy (WBRT), small-cell lung cancer (SCLC), and best supportive care (BSC).

The patient cohort and treatment groups are illustrated in Figure 1. Forty-eight percent (*n* = 141) were selected for WBRT as the initial treatment for BMs (“WBRT group”), and 52% (*n* = 153) were selected for SRT (“SRT group”). In the WBRT group, 53% (*n* = 75) were categorized as ECOG ≥ 2, 71% (*n* = 99) had ≥5 BMs, and 68% (*n* = 96) had uncontrolled ECMs. In the SRT group, 37% (*n* = 57) were categorized as ECOG ≥ 2, 2% (*n* = 3) had ≥5 BMs, and 48% (*n* = 74) had uncontrolled ECMs (Table 1).

In the WBRT group, 64% (*n* = 90) were selected for treatment with 5 × 4 Gy 98% (*n* = 88) of these completed planned treatment; 1% (*n* = 1) received 1 fraction, 1% (*n* = 1) received 4 fractions). Thirty-six percent were planned for 10 × 3 Gy 96% (*n* = 49) completed planned treatment; 2% (*n* = 1) received 2 fractions and 2% (*n* = 1) received 9 fractions. In the SRT group, 96% (*n* = 147) received 1–3 fractions and 4% (*n* = 6) received 5–10 fractions. Fraction doses varied from 5–25 Gy per fraction.

### 3.3. Overall Survival

**Figure 2:**
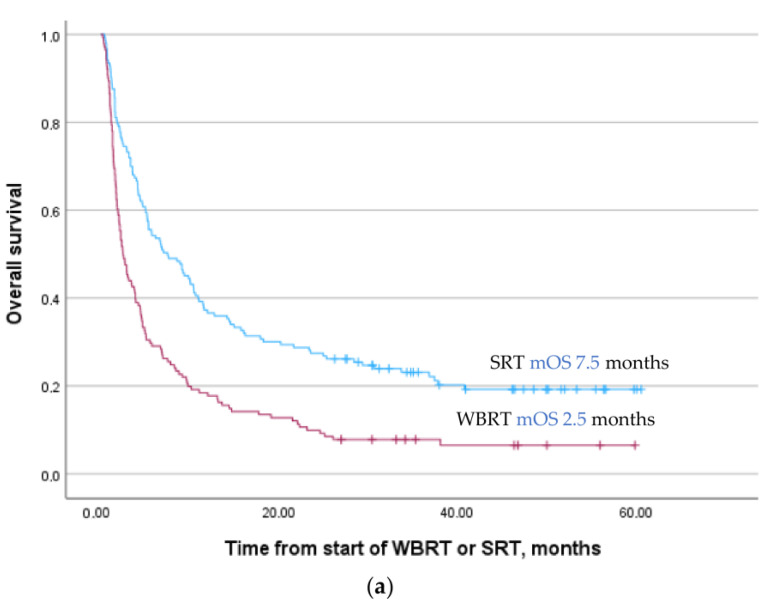

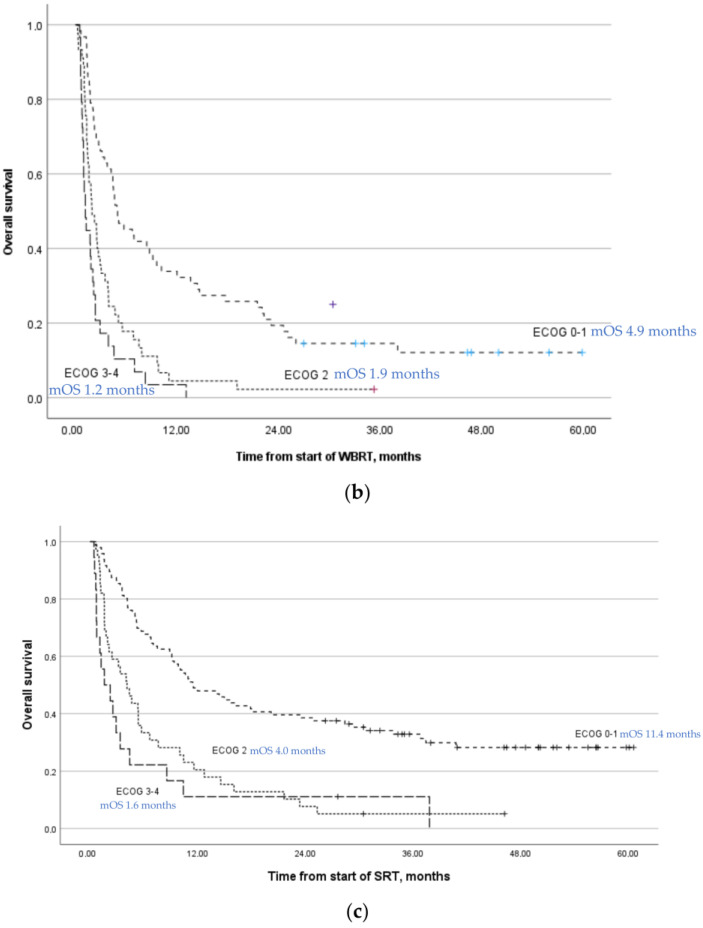
(**a**) Overall survival after radiotherapy for brain metastases, *p* < 0.001. (**b**) Overall survival after WBRT according to ECOG status, *p* < 0.001. (**c**) Overall survival after SRT according to ECOG status, *p* < 0.001. Stereotactic radiotherapy (SRT), whole brain radiotherapy (WBRT), and median overall survival (mOS).

For the total patient cohort, median OS after the start of radiotherapy was 4.6 months (95% CI 3.7–5.3); 2.5 months (95% CI 1.8–3.2) for patients selected to WBRT and 7.5 months (95% CI 4.3–10.7) for patients selected to SRT (Figure 2a). mOS for patients with ECGO 0–1, ECOG 2, and ECOG 3–4 was 10.1 months (95% CI 7.9–12.2), 3.3 months (95% CI 2.3–4.3), and 2.2 months (95% CI 1.6–2.8), respectively. Overall, 10% of the patients died within 30 days, 40% within 90 days (Table S2). Of patients who died within 90 days, (*n* = 118), 69% had ECOG ≥ 2, 70% had uncontrolled ECMs, 44% had ≥5 BMs, and 65% were treated with WBRT.

For patients in the WBRT group, mOS was equally poor for patients with ECOG 2 and ECOG 3–4: 1.9 months (95% CI 1.1–2.8) and 1.2 months (95% CI 0.7–1.6), respectively (Figure 2b). After the start of WBRT, 14% of patients died within 30 days and 54% died within 90 days, whereas 18% were alive more than 1 year after the start of treatment (Table S2). For patients in the SRT group, mOS for patients with ECOG 2 and ECOG 3–4 were 4.1 months (95% CI 2.3–5.9) and 1.6 months (95% CI 0.0–3.8), respectively (Figure 2c). After the start of SRT, 7% died within 30 days and 27% within 90 days, whereas 37% were alive more than 1 year after treatment (Table S2).

### 3.4. Lung GPA and U-RPA

Lung GPA and U-RPA analyses were performed for the total group of patients (*n* = 294). Four patients could not be included in the U-RPA calculations due to unknown ECOG status.

#### 3.4.1. Lung GPA

Overall, according to the Lung GPA scores, 32% (*n* = 97) of the patients were classified with a score of 0.0–1.0 (Table 2a,b). Sixty percent (*n* = 58) of these patients died within 90 days. Of the 118 patients that survived <90 days in our cohort, 48% (*n* = 57) were classified with a score of 0.0–1.0 (Table S3a,b).

#### 3.4.2. U-RPA

Overall, according to the U-RPA classification, 64% (*n* = 187) of the patients were classified as class 2B and 16% (*n* = 47) were classified as class 3 (Table 3). For class 2B and 3 in total, 46% (*n* = 107) of the patients died within 90 days. Of the 118 patients with survival <90 days, 61% (*n* = 71) were categorized as class 2B; 31% (*n* = 36) were categorized as class 3 (Table S4). 

### 3.5. Univariable and Multivariable Analysis

All 294 patients were included in the uni- and multivariable analyses reported in Table 4. Overall, ≥5 BMs, ECOG ≥ 2, uncontrolled ECMs, and synchronous diagnosis of BMs and lung cancer were associated with shorter overall survival (*p* < 0.05) in the multivariable analysis.

## 4. Discussion

### 4.1. Major Findings

This study has several important findings. First, patients with ECOG 3–4 were offered RT. Second, survival was poor in patients with ECOG 3–4 regardless of RT modality. Third, mOS for patients with ECOG 2 selected for WBRT was as poor as for those with ECOG 3–4. Finally, 40% of all patients died within 90 days after the start of RT, and 76% of patients with ECOG 2 selected for WBRT died within 90 days after the start of RT. These findings indicate too extensive use of RT in NSCLC patients with BMs.

### 4.2. Multivariable Analysis

Overall, in multivariable analysis, we confirmed that ECOG performance status ≥2, multiple BMs (≥5), uncontrolled ECMs, and synchronous diagnosis of BMs and lung tumor are associated with poor survival [15,23,24,25]. Available treatment options to potentially achieve control of ECMs are likely as crucial for survival as treatment of BMs, as most of these patients do not die from BMs per se. Due to improved intracerebral response rates with targeted therapy, van Schie et al. have proposed that systemic treatment options should be considered as a prognostic factor for survival [24].

### 4.3. Patients with ECOG 2–4

Patients with ECOG 2 and ECOG 3–4 selected for WBRT had equally poor survival. This was also found in a retrospective study by Frisk et al. in lung cancer patients given WBRT, in which mOS was 1.6 months in patients with a WHO score of 2, and 1.0 months in patients with a WHO score of 3–4 [15]. Natesan et al. reported a 30-day mortality of 29.9% after WBRT [26]. In that retrospective study, patients who died within 30 days had a median KPS = 50. Poor survival after WBRT is also in line with a retrospective Dutch study of NSCLC patients reporting mOS 2.7 m after WBRT [27]. Our prospective study shows the same poor survival after WBRT compared to our retrospective study published in 2021 [23]. All these studies strongly indicate PS as a prominent prognostic factor and guidelines do not recommend RT for BMs in patients with ECOG 3–4 given their poor expected survival [10].

Patients with short mOS (<3 months) do not survive long enough to expect benefits from RT and risk experiencing only the acute toxicity of radiotherapy, which results in reduced quality of life. These patients should rather be offered the best supportive care to maintain or improve quality of life near the end of life. Although the majority of the patients in this study received shortened WBRT regimens (i.e., 5 × 4 Gy), the patients spend valuable time in the hospital or as an outpatient due to the treatment. Consequently, they have less time together with family and friends at the end of life.

Guidelines use the term “poor PS” without any further specification regarding ECOG or KPS [28,29]. Based on our findings, we ask if “poor PS” should include ECOG 2 as well as ECOG 3–4, especially if the patient is not a candidate for any other intracranial tumor-directed treatments than WBRT. Many clinicians find it difficult to withhold tumor-directed therapy even if the clinical benefit is highly questionable and the guidelines are clear. That ECOG 2 could be considered a poor prognostic factor equal to that of ECOG 3–4 may be regarded as provocative. We hypothesize that when in doubt, clinicians are reluctant to categorize a patient to ECOG 3 to defend tumor-directed therapy in general, and cerebral radiotherapy in particular. Consequently, patients categorized as ECOG 2 most likely represent a heterogeneous population. This assumption is confirmed by Hollen et al. [30] who found NSCLC patients with ECOG 2 were “equally likely to have a KPS score of 60 or 70 or 80”. Therefore, if a patient categorized as ECOG 2 is not a candidate for other tumor-directed treatments, the indication for WBRT should be questioned. The findings in our study imply that for patients with ECOG 2, the number of BMs, status of ECMs, and status of primary tumor should be considered in the decision-making process. If the patient has >5 BMs and uncontrolled ECMs, the patient should most likely be offered BSC to try to sustain or improve symptoms and quality of life. Palliative care and tailored interventions improve symptom control and possibly also survival in patients with advanced NSCLC cancer with and without BMs [31]. This is also demonstrated in the QUARTZ study, showing that WBRT provides little or no clinical benefit compared to BSC alone in patients who are not candidates for surgery or SRT [13].

On the other hand, patients with ECOG 2 treated with SRT had longer mOS compared to WBRT patients. As these patients more often had good prognostic factors [100% ≤4 BMs and 49% controlled ECMs], selection bias (number of BMs, extracranial disease status, and patient performance status) is the most plausible explanation for the prolonged survival for SRT patients and not the treatment modality per se.

### 4.4. Lung GPA and U-RPA

Prognostic tools might help to determine which patients would live long enough to benefit from tumor-directed therapy, and which will benefit more from best supportive care (BSC) alone. To the best of our knowledge, this prospective study is the first to validate the U-RPA prognostic tool in a real-life cohort of NSCLC patients with BMs treated with RT only. Lung GPA includes molecular markers while U-RPA does not, and U-RPA encompasses a limited number of variables and information. This makes U-RPA applicable at an earlier stage, e.g., at the initial presentation of brain metastases. In our opinion, Lung GPA and U-RPA complement each other and hence constitute the basis for our decision to use these two prognostic tools, regardless of the frequency of the mutations, which is also low because patients treated with targeted therapies as first-line treatment were not included in this present study.

Prognostic tools may be incorporated into electronic medical journals. Still, a shared decision-making process involving the treating physician, the patient, and the patient’s next of kin is of the utmost importance to discuss the patient’s symptoms, preferences, and the total situation in relation to the viable treatment options and their side effects. Prognostic tools can only be a supplement in this regard.

## 5. Strengths and Limitations

Strengths of our study include the prospective design with a large sample size of a real-life cohort with few exclusion criteria, thereby minimizing selection bias. The results are generally applicable, representing daily clinical practice at several oncological centers.

This was a multicenter study recruiting patients from several hospitals. Although the majority of the patients were included at a tertiary referral university hospital, most patients were referred from local hospitals. Therefore, the patients represent a real-life patient cohort.

ECOG performance scale is widely used in daily clinical work but with an acknowledged unavoidable inter-rater variability.

The higher number of patients in the best prognostic groups of Lung GPA and U-RPA in randomized trials is probably due to inclusion criteria in such trials.

## 6. Conclusions

This study found too extensive use of RT in NSCLC patients with BMs and poor PS. Patients with ECOG 3–4 should not be offered RT, regardless of modality. If not candidates for surgery or SRT, patients with ECOG 2 should be carefully considered before being offered WBRT, especially if ECMs are not under control or without prospects of obtaining control. Patients estimated to live less than three months after the BM diagnosis should rather be offered BSC alone for the best care near the end of life. SRT may be justified for selected patients with ECOG 2.

## Figures and Tables

**Table 1:** Characteristics of all patients at inclusion.

	All Patients *n* 294 (%)	WBRT *n* 141 (%)	SRT *n* 153 (%)	*p*-Value WBRT vs. SRT
**Age**				0.430
<70	152 (52)	76 (54)	76 (50)	
≥70	142 (48)	65 (46)	77 (50)	
**ECOG**				0.009
ECOG 0–1	158 (54)	62 (44)	96 (63)	
ECOG 2	85 (29)	46 (32)	39 (25)	
ECOG 3–4	47 (16)	29 (21)	18 (12)	
Unknown	4 (1)	4 (3)		
**Histology**				0.092
Adenocarcinoma	216 (73)	111 (79)	105 (69)	
Squamous cell carcinoma	44 (15)	14 (10)	30 (20)	
Others *	34 (12)	16 (11)	18 (11)	
**Number of BMs**				<0.001
1	89 (30)	9 (6)	80 (52)	
2–4	100 (34)	30 (21)	70 (46)	
≥5	102 (35)	99 (71)	3 (2)	
Unknown	3 (1)	3 (2)		
**EGFR mutation**				0.846
Yes	18 (6)	9 (6)	9 (6)	
No/unknown	276 (94)	132 (94)	144 (94)	
**ALK translocation**				0.929
Yes	4 (1)	2 (1)	2 (1)	
No/unknown	299 (99)	139 (99)	153 (99)	
**PD-L1 positive**				0.009
Yes	153 (52)	62 (44)	91 (59)	
No/unknown	141 (48)	79 (56)	62 (41)	
**Clinical status primary tumor**				0.305
Primary tumor controlled/removed	86 (30)	37 (26)	49 (32)	
Progressive/not evaluated	55 (18)	31 (22)	24 (16)	
Synchronous diagnosis of BMs and lung tumor	153 (52)	73 (52)	80 (52)	
**Extracranial metastases**				<0.001
Controlled	124 (42)	45 (32)	79 (52)	
Uncontrolled	170 (58)	96 (68)	74 (48)	

Whole brain radiotherapy (WBRT) and stereotactic radiotherapy (SRT). * Large cell carcinoma, unknown, others.

**Table 2:** (**a**) Patients with adenocarcinomas classified according to Lung GPA. (**b**) Patients with non-adenocarcinomas classified according to Lung GPA. * Predicted mOS m (IQR) according to the original Lung GPA.

(**a**)							
	**Total Cohort Adenocarcinoma**		**WBRT**		**SRT**		**Lung GPA**
**Score** **Lung GPA**	**Actual mOS m** **(IQR)**	***n* = 216 (%)**	**Actual mOS m** **(IQR)**	***n* = 110 (%)**	**Actual mOS m** **(IQR)**	***n* = 104 (%)**	Predicted mOS m (IQR) *
0.0–1.0	3.5 (2, 9)	80 (37)	3.2 (2, 7)	62 (56)	6.8 (2, 12)	18 (17)	6 (2, 13)
1.5–2.0	6.9 (3, 23)	96 (44)	4.3 (3, 15)	43 (39)	10.2 (3, 38)	53 (51)	15 (5, 38)
2.5–3.0	25.1 (7, NR)	38 (18)	38.2 (6, NR)	5 (5)	25.1 (8, NR)	33 (32)	30 (12, NR)
3.5–4.0		2 (1)					52 (25, 69)
(**b**)							
	**Total Cohort Non-adenocarcinoma**		**WBRT**		**SRT**		**Lung GPA**
**Score** **Lung GPA**	**Actual mOS m** **(IQR)**	***n* = 78 (%)**	**Actual mOS m** **(IQR)**	***n* = 30 (%)**	**Actual mOS m** **(IQR)**	***n* = 48 (%)**	**Predicted mOS m (IQR)**
0.0–1.0	2.2 (2, 4)	17 (22)	2.4 (2, 4)	13 (43)	1.4 (1, 2)	4 (8)	2 (1, 49)
1.5–2.0	3.7 (2, 7)	28 (36)	2.9 (2, 4)	10 (33)	5.5 (2, 7)	18 (38)	5 (3, 12)
2.5–3.0	5.2 (3, 12)	27 (35)	2.7 (2, 5)	6 (20	5.7 (2, 16)	28 (44)	10 (4, 21)
3.5–4.0	4.6 (3, 24)	6 (7)		1 (3)	17.1 (5, 24)	5 (10)	19 (8, 33)

Lung Graded Prognostic Assessment (GPA), not reached (NR), whole brain radiotherapy (WBRT), and stereotactic radiotherapy (SRT).

**Table 3:** Patients classified according to U-RPA. * Four patients were not included due to missing ECOG status.

U-RPA	Total Cohort *		WBRT		SRT		U-RPA
	Actual mOS Months (95% CI)	*n* = 290 (%)	Actual mOS Months (95% CI)	*n* = 137 (%)	Actual mOS Months (95% CI)	*n* = 153 (%)	Predicted mOS Months
Class 1	17.1 (0–42.3)	16 (6)	22.3 (0.0–46.7)	8 (6)	9.5 (0.0–26.5)	8 (5)	28.1
Class 2							
A	14.9 (4.1–25.8)	40 (14)	4.3 (0.0–13.7)	12 (9)	19.7 (0.0–49.9)	28 (18)	14.7
B	5.6 (4.2–7.0)	187(64)	3.4 (2.7–4.2)	88 (64)	7.5 (4.5–10.4)	99 (65)	7.6
Class 3	2.2 (1.6–2.8)	47 (16)	2.0 (1.3–2.6)	29 (21)	2.5 (1.4–3.6)	18 (12)	3.3

Updated Recursive Partitioning Analysis (U-RPA), whole brain radiotherapy (WBRT), and stereotactic radiotherapy (SRT).

**Table 4:** Overall survival after initial RT.

All Patients *n* 294					
	*n*	Unadjusted HR (95% CI)	*p*-Value	Adjusted HR (95% CI)	*p*-Value
**Age**					
<70	152	1		1	
≥70	142	1.3 (1.0–1.6)	0.053	1.3 (1.0–1.6)	0.082
**ECOG**					
ECOG 0–1	158	1		1	
ECOG 2	85	2.5 (1.9–3.4)	<0.001	2.3 (1.7–3.1)	<0.001
ECOG 3–4	47	4.0 (2.8–5.7)	<0.001	3.8 (2.6–5.5)	<0.001
Unknown	4	1.3 (0.4–4.0)	0.685	0.8 (0.2–2.5)	0.674
**Histology**					
Adenocarcinoma	216	1		1	
Squamous cell carcinoma	44	1.4 (1.0–2.0)	0.055	1.4 (0.9–1.9)	0.105
Others *	34	1.7 (1.2–2.5)	0.006	1.7 (1.1–2.6)	0.009
**Number of BMs**					
1	89	1		1	
2–4	100	1.1 (0.8–1.5)	0.658	1.4 (1.0–1.9)	0.052
≥5	102	1.6 (1.1–2.1)	0.005	1.5 (1.1–2.2)	0.012
Unknown	3	4.5 (1.4–14.5)	0.011	5.3 (1.5–17.9)	0.008
Mutation/PD-L1 positive **					
Yes	172	1		1	
No	122	1.3 (1.0–1.6)	0.051	1.1 (0.8–1.4)	0.505
**Clinical status primary tumor**					
Primary tumor controlled/removed	86	1		1	
Progressive/not evaluated	55	1.4 (1.0–2.1)	0.044	1.3 (0.9–1.9)	0.214
Synchronous diagnosis of BMs and lung tumor	153	0.9 (0.7–1.2)	0.399	0.7 (0.5–1.0)	0.045
**Extracranial metastases**					
Controlled	124	1		1	
Uncontrolled	170	1.7 (1.3–2.1)	<0.001	1.7 (1.3–2.3)	<0.001

* Large cell carcinoma, unknown, others. ** Patient harboring EGFR mutation, ALK alteration, or PD-L1 positive tumor.

## Data Availability

The data from this study may be made available upon request to the corresponding author who will review and seek approval from study investigators.

## Supplementary Materials

The following supporting information can be downloaded at: https://www.mdpi.com/article/10.3390/cancers16081486/s1, Table S1: STROBE Statement—Checklist of items that should be included in reports of *cohort studies*; Table S2: Overall survival after initial RT; Table S3: (a) Patients with adenocarcinoma with survival less than 3 months (*n* = 79) classified according to Lung GPA, (b) Patients with non-adenocarcinoma with survival less than 3 months (*n* = 39) classified according to Lung GPA; Table S4: Patients with survival less than 3 months (*n* = 118) classified according to U-RPA.
